# Monthly Summary

**Published:** 1877-03

**Authors:** 


### MONTHLY SUMMARY.

*Chemical Porcelain.*—A new kind of pottery ware has been invented by Mr. R. W. Wallace, who gives the material the name of "Chemical Porcelain."

This porcelain appears to be a happy mixture of natural products, fire clay, etc., which makes ware of extraordinary quality. As it has been submitted to Professor Noad, we shall give his opinion of its merits, without comment on our parts :

" In this invention the kaolin in porcelain is replaced by a mineral material, and besides being remarkably strong, it will resist the most extreme variations of temperature. I selected seven vessels of different sizes and shapes, and saw them thrown without any previous annealing, into a furnace, where they were allowed to remain till white hot, when they were removed and thrown into cold water. They all stood the crucial test perfectly.

I examined the vessels when cold, and found them all perfectly sound and unaltered.

I am not acquainted with any porcelain which could be subjected to this ordeal with such satisfactory results.

I saw concentrated oil of vitriol boiled for half an hour or more in a double-bent tube made of this material. Whilst boiling, I caused cold water to be thrown into the tube ; this, of course, caused furious boiling, almost amounting to explosion. I then had the tube plunged into cold water, but it remained perfectly sound. I afterwards examined the acid, but found in it neither silica nor albumina. I am satisfied, therefore, that this new compound may be substituted for platinum in the construction of vessels for the continuous concentration of sulphuric acid ; and, in fact, for every other chemical purpose for which platinum and silver are now considered indispensable *e. g.*, stills and worms for pharmaceutical purposes, for refining nitric acid and other acts, for pots for the manufacture of phosphorus, iodine, etc., for evaporating dishes, retorts, etc., which are now made of Berlin or Meissen ware. The cost of the production of articles of this compound bear so slight a proportion to that of the platinum and silver vessels now in use, that the saving in the original cost of the porcelain will be not only very great, but a large saving in the shape of interest upon the money sunk in the purchase of these metals, will be effected, and also as there is no action on the porcelain, the chemicals produced would be much purer; as, for instance, sulphuric, citric and tartaric acid would contain no salts of lead, which would be of great advantage to the consumer."

There is no doubt this material supplies a want long felt by chemical manufacturers: and if, as we believe it is intended, a company is formed to work the patent, it cannot fail to be a success.—*Journal of Materia Medica.*

---

*A Fatal Case of Parotitis.*—Owing to the unusual circumstance of a fatal termination of the above disease, I think it of sufficient importance to send it for publication.

On the 10th of January, 1876, I was called in consultation in the case of N. S., a lad seventeen years of age, by occupation a farmer, of good constitution, having always enjoyed good health except about nine months ago he had an attack of epilepsy, from which, however, he had fully recovered.

First, he complained of considerable headache. The next day he rode several miles through mud and rain to the village on business, and returned feeling quite unwell, and was taken with fever, which was followed by intense vomiting. In a few days his right jaw began to swell, and from an irritable molar tooth in the inferior maxilla, which had caused him trouble previously, an abscess formed and opened internally, discharging profusely. The swelling remained for several days, when it began to subside, and swelling commenced in the opposite parotid gland, involving the submaxillary and sublingual glands, and extended all over the left side of the face and head, closing the eye almost completely and locking the jaw so firmly that liquid nourishment was administered with great difficulty. When I saw the patient first, about two weeks after the first attack, the swelling had just commenced on the left side, and he was somewhat delirious. The pulse ranged from 84 to 90 and the temperature in the axilla 100° to 101°. The discharge still continued from the ulcerated tooth on the right side, and it being impossible to place the patient in a position for the pus to discharge itself through the mouth, he swallowed more or less in his delirium. In a few days there were symptoms of pyæmic poisoning, which resulted in his death.—*John Vite, M. D., in American Medical Bi-Weekly.*

*Chloral Plaster in Neuralgia.*—Dr. Solari, of Marseilles, says the *Medical Examiner*, recommends the chloral plaster as an excellent application in cases of neuralgia and of nervous pains resulting from exposure to cold. The plaster is easily prepared by powdering the chloral over a common pitch plaster, one to two scruples of chloral for every four square inches of the plaster. Care is taken not to incorporate the chloral with the pitch. It is applied for twenty-four to forty-eight hours; when removed, the skin is found covered by a number of small vesicles; these are opened, and the part then covered with a cerate dressing. Generally speaking, it will be found that the pain has disappeared before the vesicles heal. Dr. Solari states that numerous cases of lumbago, intercostal and other forms of neuralgia, et.c, have been rapidly cured by this simple method. —*Nashville Journal of Medicine and Surgery.*

---

*Choice of Sedative for the Young or Aged.*—Dr. Stokes (Guy's Hospital Reports for 1876) says: "If we propose giving a sedative to the very old or very young, we must be cautious, especially in using any of the preparations of opium, as with them they are not only prepotent, but often cumulative their in effects. As a consequence of this, some years past I have trusted almost entirely to sedatives other than opiates in treating children in their first septennate, and I have seen no reason to believe that any want of success has ensued from this exclusiveness. That such a precautionary measure is not altogether uncalled for has been impressed upon me by my experience of the method of medication adopted by the more ignorant (including nurses and nursery-maids,) whose frequent habit is to increase the prescribed dose several-fold, or to repeat it with undue persistence if it should fall short of the expected effect; with what result may be conceived when two or three minims of laudanum have been ordered for an infant. With potassium bromide and conium for the various morbid conditions incidental to teething; chloroform for administration during the paroxysm of a convulsive attack; chloral for those derangements in which insomnia is the prevailing symptom; aconite for inflammations, fevers and feverishness generally; belladonna and hyoscyamus for many visceral disorders of a painful and obstinate nature; and combinations of these and other drugs to soothe coughs and the innumerable aches and pains of neuralgic, myalgic, or rheumatic origin—to say nothing of a host of external sedative applications, many of which are very potent—we need be under no apprehension lest we should be incapable of coping with the assaults of disease in children as effectually as we could do with one more weapon in our repertory."

*For the Aged.*—"If we think fit to employ opium as an anodyne or hypnotic with those who have attained to or are on the high road to second childhood, it is judicious to combine chloral and spirit of chloroform with it; the opium being prescribed in excess when pain, the chloral when restlessness, and the spirit of chloroform when cramp predominate; and the quantities of several ingredients need not be large, as each of them intensifies the effect of the others. The addition of from ten to twenty minims of the tincture of Indian hemp, a very invigorating soporific, to such a mixture as the above is most serviceable in dealing with a heart enfeebled by advanced age or exhausting illness; and in thus prescribing it I have invariably met with an exemption from the distressing symptoms which sometimes result from the oppressive action of opiates on the respiratory system."—*Louisville Med. News.*

---

*Nervous Sensitiveness.*—"A comparison of the present time with a quarter or half century ago."

He finds that nervous senstiveness generally, has increased; that our fathers were not affected by extremes of temperature as we are; that the coarser articles of diet, like pork, etc., were better borne; that opium was well borne, while the more sensitive modern requires the bromides and chloral in place of it, for producing sleep; and that idiosyncracies in the direction of certain substances are far more common now than then.

By a comparison of the present time with the middle ages, and the few centuries preceding the nineteenth century.

The functional nervous affections were represented by chorea and epidemics of the emotional type of hysteria. He considers the evidence in favor of an increase in the number of the insane, in recent periods, to be overwhelmingly in its favor.

By comparing the nervous status of the women of our time with that of her sex in past times.

"The infinite array of aches and pains that follow in the path of the modern woman needs not statistics to prove its presence, even if statistics could be obtained. The diminution in the increase of the population in the better classes of American society is another fact to be noted. Also that female inebriates are not at all uncommon. The climate of this country, especially that of the Northern and Eastern sections, bears an intimate relation to the nervousness which characterizes a large proportion of the better classes. The remarkable dryness of the atmosphere and the rapid alternations of extreme heat and extreme cold, doubtless have much to do in causing nervous exhaustion. This state of affairs progressively diminishes as one goes south. Functional nervous disorders likewise diminish as the Gulf is approached.

He claims that sensitiveness of the nervous system is an aid to longevity, and that brain-workers are, as a rule, longer-lived than the muscle-workers. "Probably no great class of people in the world live longer than the professional and business men of America—the very class among whom these nervous disorders are so often found, the class that supplies the victims for our inebriate asylums. * * * * * Notably in our country, where nervous sensitiveness is seen in its extreme manifestations, the majority of brain-workers are not safe so long as they are in the habit of even moderate drinking."—*Abstract from Address of Dr. G. M. Beard, in American Inebriety Journal.*

---

*Injection of Simple Cold Water in Neuralgia.*—It will be remembered that Dr. Leybert, of France and others, extolled the use of hypodermic injections of pure water in neuralgia. In the London *Medical Times and Gazette,* Dr. Henry Lawson, himself a sufferer from sciatica, details his own experience as follows:—

In conclusion I must make a few remarks on the treatment of sciatica and lumbago by injections, simply of ordinary cold water. In this mode of operation I have not the smallest faith; firstly, because I have never seen an instance of it myself, though I have attempted it in various cases; secondly, because I have tried it upon myself without the least effect. Possibly the reader will say that the reason in the latter case was that "You knew the difference between the two cases (viz., that in which morphia had been administered, and that in which it had not been given,) and thus that the mental effect was prevented." But this was not so. I especially took care that the syringe was filled by a second person, who completely concealed from me whether mere warm water, or water containing morphia, had been employed. Yet in each case I was enabled to tell exactly, in the course of from five to eight minutes, whether the morphia or simply water had been used. Indeed, I could detect the difference at once; before the syringe had been well withdrawn; but I waited after each injection, in order to be certain of the actual state of the case, and in no instance did I fail in my judgment.

It has been the same with patients who have been accustomed to the use of morphia subcutaneously, all of whom can tell, in the course of a few minutes, whether the injection has been morphia or water; nay, they can soon tell you whether you have given them the ordinary or a larger dose.—*London Med, Times.*

*Violent Pain—Its Effects on Animals and Men.*—Mantsgazza, (*Hirsch Jahresbericht,*) by experiments proved that in animals violent pain produced loss of appetite, dyspepsia, nausea, arrest of the digestive process, vomiting and diarrhœa, and if continued, great weakness and considerable loss of weight and greater sensitiveness to all injurious agencies In man we find that severe neuralgic pain produces dyspepsia, want of appetite, coated tongue, pallor and anæmia, weak pulse, cold extremities, depressed, excitable states of the system, great sensitiveness to external injurious influences.—*Jour. Materia Medica.*

---

*The Poisonous Action of Salicylic Acid.*—Professor Abelin, of Stockholm, states that, in children, salicylic acid, when given in a dose large enough to produce a lowering of temperature from 2° to 4°, was badly tolerated, and produced serious symptoms and great depression. In a dose of 0.8 to 1 gramme (*i. e.* about 12⅓ to 15⅘ grains) it acted, in an infant at the breast, as a violent poison. It has also a very irritant action on the mucous membranes of the mouth and pharynx. This action prevents the child from swallowing and sucking. It produces rapidly a lowering of more than 5° Fahr., a considerable amount of collapse, irregular respiration, altered skin functions, and a strong fluxion of blood to certain viscera. The following case is given as an example: A child at the breast, aged four months, up to that time healthy, became febrile from vaccinnation. Temperature in the morning 104° ; dejecta natural. Three vaccine bullæ beginning to suppurate. Ordered fifteen and two-fifths grains of salicylic acid, to be divided into three portions. A good part was lost in struggling, so that not more than about twelve grains were actually taken. The child became rapidly restless, and unable to suck. The mouth and pharynx became coated with a thin white deposit. The child could not swallow ; the pharynx got full of mucus. Respiration was difficult and rattling ; there was obviously an obstacle to the entrance of air into the larynx. The face looked pinched ; the eyes sunken. The temperature fell rapidly, and in two hours it was only 98.6°. The child got rapidly worse ; the pulse small and unequal—160 ; face pale yellow. There was inability to swallow, or to utter a sound, and extreme restlessness. Death followed in a few days.

To sum up, Dr. Abelin is of opinion that in young children the use of salicylic acid must be very restricted. In a dose of twelve to fifteen grains it acts as a corrosive poison ; in smaller quantities it lowers the temperature without acting beneficially on the course and symptoms of the malady.—*London Med. Times and Gazette.*

*Spasmodic Contraction of Muscles Treated by Excision of Nerves.*—At a recent meeting of the Royal Medical and Chirurgical Society, Mr. W. Spencer Watson read notes of the case of a young lady, aged nineteen years, who was the subject, when nine months old, of so-called infantile paralysis. The right arm and right leg had ever since been contracted at the wrist and ankle. The deformity of the foot, however, had been remedied by division of the tendo-Achillis in infancy, and the patient had been able to walk with a limp ever since. The right arm, forearm, and hand, had, beside the permanent contraction of the flexors, been more recently subject to violent spasmodic movements, which had produced exhaustive debility and a tendency to hysterical paroxysms. After the failure of general remedies and of tenotomy of the more prominent flexors, Mr. Watson divided and excised a portion of the median nerve at the elbow. This relieved the muscular spasm to some extent, and somewhat reduced the previously heightened temperature of the limb. The deep flexors and adductors, however, continued to be subject to violent spasmodic movements, and the wrist continued permanently flexed. Mr. Watson therefore, with Sir William Gull's sanction, excised a portion of the ulnar nerve, behind the inner condyle of the humerus. The contracted muscles were at once released, and the forcibly adducted thumb and little finger set free. The total effect of all the operations has been decidedly beneficial to the patient, but she still complains of pain in those parts now paralyzed as to sensation and motion. The operations of neurectomy and tenotomy were undertaken in this case, on the hypothesis that the original central lesion was become secondarily aggravated by the constant peripheral irritation, either associated with, or caused by, the convulsive muscular movements. The primary mischief was central; the peripheral irritation reacted upon the centre. A vicious circle of cumucative nerve-irritation was thus established. The object of the operations was to break the continuity of that circle.—*Med. & Surg. Reporter.*

---

*Tincture of Aconite Root to Stop the Toothache.*—Dr. Stevens, in the *Progres Dentaire*, advises the use of the tinct. aconit. rad. in inflammations of the gums. In case an alveolar abscess is imminent, dry the gum with cotton and apply a drop of the tinct. of aconite. After the extraction of a tooth, one or two drops applied on a tampon of cotton produces an immediate relief from pain.—*Canada Medical.*

